# Mechanisms of Polycomb group protein function in cancer

**DOI:** 10.1038/s41422-021-00606-6

**Published:** 2022-01-19

**Authors:** Victoria Parreno, Anne-Marie Martinez, Giacomo Cavalli

**Affiliations:** Institute of Human Genetics, UMR 9002, CNRS–University of Montpellier, Montpellier, France

**Keywords:** Cancer, Molecular biology

## Abstract

Cancer arises from a multitude of disorders resulting in loss of differentiation and a stem cell-like phenotype characterized by uncontrolled growth. Polycomb Group (PcG) proteins are members of multiprotein complexes that are highly conserved throughout evolution. Historically, they have been described as essential for maintaining epigenetic cellular memory by locking homeotic genes in a transcriptionally repressed state. What was initially thought to be a function restricted to a few target genes, subsequently turned out to be of much broader relevance, since the main role of PcG complexes is to ensure a dynamically choregraphed spatio-temporal regulation of their numerous target genes during development. Their ability to modify chromatin landscapes and refine the expression of master genes controlling major switches in cellular decisions under physiological conditions is often misregulated in tumors. Surprisingly, their functional implication in the initiation and progression of cancer may be either dependent on Polycomb complexes, or specific for a subunit that acts independently of other PcG members. In this review, we describe how misregulated Polycomb proteins play a pleiotropic role in cancer by altering a broad spectrum of biological processes such as the proliferation-differentiation balance, metabolism and the immune response, all of which are crucial in tumor progression. We also illustrate how interfering with PcG functions can provide a powerful strategy to counter tumor progression.

## Introduction

Polycomb Group (PcG) proteins have first been described as main players in cellular memory known to maintain embryonic chromatin landscapes in a repressed transcriptional state throughout development. Counterintuitively, PcG proteins then appeared to be able to regulate the transcription of developmental genes involved in a wide range of highly dynamic biological processes such as differentiation, stem cell plasticity or cell cycle progression.^1,2^ In addition, mutations or dysregulations of PcG proteins have been extensively described in cancer.^3^ Knowing the importance of PcG proteins in transcriptional regulation, it was not surprising to find a correlation between modification of PcG activities and tumorigenesis. However, an early demonstration of a causal link between the ability of PcG complexes to promote or inhibit the transcription of oncogenes or tumor suppressor genes, respectively, has paved the way for work aimed at studying the different mechanisms by which PcG complexes are involved in the generation and the evolution of cancer cells.

Here, we first describe the molecular mechanisms underlying the recruitment and function of PcG proteins in gene regulation during normal development. We then review the involvement of Polycomb complexes in cancer, highlighting PcG-dependent disturbances of epigenetic processes in tumorigenesis. Next, we focus on the description of the latest discovered mechanisms linking Polycomb to cancer. PcG proteins have been extensively studied in hormone-dependent cancers where hormone-receptors interact directly with PcG proteins, modifying the transcriptional landscape of the affected cells. Furthermore, PcG proteins have been described as capable of modulating the metabolism and the immune response of the tumor microenvironment, both being hallmarks of cancer. Next, we focus on a new area of research involving mutated histones, also known as oncohistones, and discuss how these mutations can impact PcG behaviour in a tumoral context. Finally, we explain how PcG proteins are able to confer a non-genetic drug-resistance underlying the importance of epigenetics in cancer.

## PcG Proteins

PcG proteins are highly conserved throughout metazoan evolution and are essential players in cellular identity. In *Drosophila melanogaster*, mutations in the *Polycomb* gene induce embryonic transformation of anterior segments into posterior segments by inducing ectopic expression of homeotic (Hox) genes.^4,5^ Subsequent work identified other mutations triggering derepression of Hox genes, leading to the identification of several genes that were defined as members of the Polycomb group. PcG proteins form two main epigenetic complexes, the Polycomb Repressive Complex 1 and 2 (PRC1 and PRC2), which were later identified as transcriptional regulators targeting a large number of genes in genome-wide studies.^6^

**Fig. 1:**
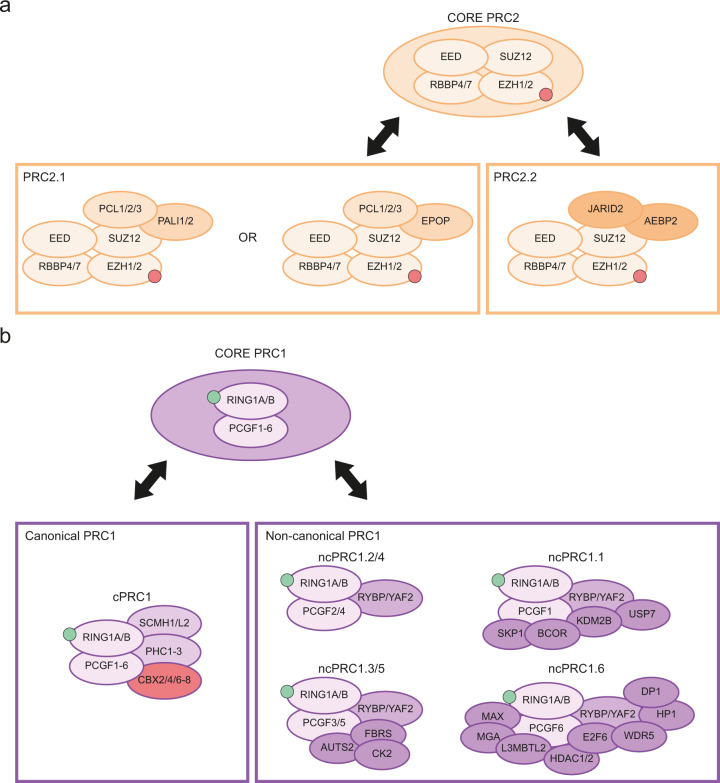
Composition of PcG proteins in mammals. **a** PRC2 can be sub-divided into PRC2.1 and PRC2.2. The core PRC2 with PCL1/2/3 and the PALI1/2 or EPOP subunits compose the PRC2.1 complex. The association between the core PRC2, JARID2 and AEBP2 constitute PRC2.2. **b** PRC1 complex can be sub-divided into two groups of complexes, namely cPRC1 and ncPRC1. cPRC1 is composed of RING1A/B associated with PCGF2/4 and CBX2/4/6–8. ncPRC1 is composed of either RYBP or YAF2 associated with one of the six PCGF proteins. The graphical representation of each complex is schematic and not aimed to represent the size, shape and relative position of the various subunits. AEBP2, Adipocyte enhancer-binding protein 2; AUTS2, Autism susceptibility candidate 2; BCOR, BCL6 corepressor; CBX2/4/6–8, Chromobox 2/4/6–8; CK2, Casein kinase 2; DCAF7, DDB1 and CUL4 associated factor 7; DP1, E2F dimerization partner 2; E2F6, E2F transcription factor 6; EED, Embryonic ectoderm development; EPOP, Elongin B/C and PRC2-associated protein; EZH1/2, Enhancer of zeste homolog 1/2; FBRS, Fibrosin; HDAC1/2, Histone deacetylase 1/2; HP1, Heterochromatin protein 1 gamma (here labeled as HP1, also named CBX3); JARID2, Jumonji AT rich interactive domain 2; KDM2B, Lysine demethylase 2B; L3MBTL2, Lethal(3) malignant brain tumor-like protein 2; MAX, Myc associated factor X; MGA, MAX gene associated protein; PALI1/2, PRC2 associated LCOR isoform 1/2; PCGF1–6, Polycomb group finger 1–6; PCL1–3, Polycomb like protein 1–3; PHC1–3, Polyhomeotic-like protein 1–3; RBBP4/7, Retinoblastoma binding protein 4/7; RING1A/B, Really interesting new gene 1B/A; RYBP, RING1 and YY1 binding protein; SCMH1/2, Sex comb on midleg homolog 1/2; SKP1, S-phase kinase associated protein 1; SUZ12, Suppressor of zeste 12 protein homolog; USP7, Ubiquitin specific peptidase 7; YAF2, YY1-associated factor 2; WDR5, WD repeat domain 5.

PRC2 is composed of the Embryonic Ectoderm Development (EED), Suppressor of Zeste 12 Homolog Protein (SUZ12) and Enhancer of Zeste Homolog 1/2 (EZH1/2) core constitutive subunits (Fig. 1a). EZH1/2 have a Su(var)3–9, Enhancer-of-zeste and Trithorax (SET) domain with a histone methyltransferase activity that mono-, di- or tri-methylates the lysine 27 of the histone H3 (H3K27me1/2/3).^7^ PRC2 can be divided into two sub-complexes, namely PRC2.1 and PRC2.2, characterized by the association with specific accessory proteins. PRC2.1 contains one of the three paralogous Polycomb-like (PCL) proteins PCL1/2/3, also known as PHF1, MTF2, PHF19 respectively, as well as PRC2-Associated LCOR Isoform 1/2 (PALI1/2) or Elongin B/C and PRC2-associated Protein (EPOP). In addition of the core subunits, PRC2.2 contains Jumonji and AT-Rich Interaction Domain containing 2 (JARID2) and Adipocyte Enhancer-Binding Protein 2 (AEBP2). Some PRC2 co-factors can have a negative impact on PRC2 methyltransferase activity. The Catalytic Antagonist of Polycomb (CATACOMB)-PRC2 variant presents a decrease in PRC2 enzymatic activity. Indeed, the *CATACOMB* (also known as *EZHIP*) gene is poorly expressed in physiological conditions, except in gonads,^8^ due to hypermethylation of its CpG islands (CGIs).^9^ While CATACOMB–PRC2 association does not impact PRC2 recruitment to chromatin, it lessens its ability to associate with sub-stochiometric co-factors that would otherwise enhance its enzymatic activity.^8^

PRC1 members form an even more diversified combination of variant complexes (Fig. 1b), which can be subdivided into canonical PRC1 (cPRC1) and non-canonical PRC1 (ncPRC1) complexes that all share a core PRC1 comprising one of six Polycomb Group Ring Finger 1–6 (PCGF1–6) proteins and RING1A/B, an E3 ubiquitin ligase catalyzing the mono-ubiquitination of lysine 119 of histone 2A (H2AK119ub in mammals or H2AK118ub in flies).^10,11^ cPRC1.2 and cPRC1.4 are respectively formed by PCGF2 or PCGF4 (also known as MEL-18 or BMI-1), RING1A/B and Sex Comb on Midleg Homolog 1/Like 2 (SCMH1/L2), and can be distinguished from ncPRC1s by the presence of one of Chromobox 2/4/6–8 (CBX2/4/6–8) proteins as well as one of the Polyhomeotic Homolog 1–3 (PHC1–3).^6,12^ In addition to RING1A/B and a PCGF1–6 protein, ncPRC1complexes assemble around RING1 and YY1-Binding Protein (RYBP) or YY1-Associated Factor 2 (YAF2) proteins, which are mutually exclusive homologous proteins able to bind to the same site on the C-terminal domain of RING1B.^13,14^ Moreover, the ncPRC1 complexes can be further classified by the identity of their PCGF subunit (PCGF1 for PRC1.1, PCGF2 for PRC1.2 and so on). Genome-wide analysis demonstrated that each PRC1 complex has its own chromatin targeting profile suggesting that the recruitment of cPRC1 and ncPRC1 depends on their differential compositions that in turn could contribute to pleiotropic functions.^15^

## Molecular mechanisms modulating prc1 and prc2 recruitment

**Fig. 2:**
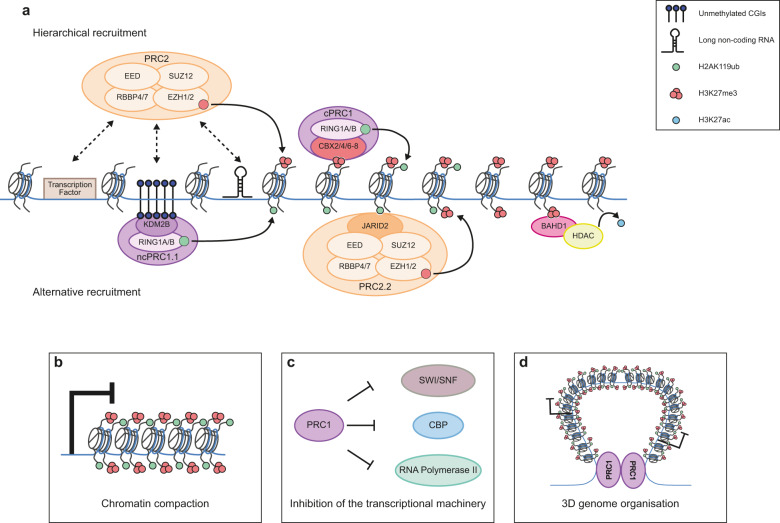
Polycomb recruitment and action on target genes. **a** First described in *Drosophila melanogaster*, the original pathway of PcG recruitment relies on two sequential steps. First, PRC2 is recruited to chromatin and deposits the repressive H3K27me3 mark via its EZH1/2 subunit. The repressive mark is then recognized by the CBX2/4/6–8 chromodomain, a subunit of cPRC1. Lastly, RING1A/B deposit the ubiquitination on H2AK119 (in *Drosophila*, H2AK118). While in *Drosophila melanogaster* PRC2 recruitment depends on specific transcription factors binding to PREs, in mammals PRC2 recruitment can occur at CGIs or depends on transcription factors or lncRNAs. More recent data suggest an alternative recruitment pathway, in which ncPRC1 complexes are recruited in a KDM2B-dependent manner which deposits the H2AK119 ubiquitination mark. In turn, this mark is recognized by the JARID2 subunit of PRC2.2. Furthermore, PRC2.1 binds the same targets via PCL1/2/3 proteins. Finally, cPRC1 is recruited via CBX2/4/6–8-mediated recognition of H3K27me3. Moreover, the new PcG proteins BAHD1 and BAHD2 have also been found to recognize the H3K27me3 repressive mark. Their interactions with HDACs generate a hypoacetylated chromatin state which participates in transcriptional silencing. **b** Chromatin compaction impairs the transcription of target genes. **c** PcG-mediated silencing depends on the inhibition of the transcriptional machinery while the repressive PRC2 and PRC1 marks are necessary to inhibit the deposition of active histone marks. **d** PRC1 participates in the higher-order 3D chromatin organization via its PHC subunits. The SAM domain of PHC-PRC1 is able to oligomerize which results in the maintenance of the transcriptionally repressed state. BAHD1, Bromo adjacent homology domain containing 1; CBP, CREB binding protein; HDAC, Histone deacetylase; RNA Pol II, RNA Polymerase II; SWI/SNF, Switch/sucrose non-fermentable.

An important feature of the PRC1 and PRC2 core subunits is the absence of sequence-specific DNA-binding domains that would allow their direct recruitment to their target genes. PcG-mediated gene regulation therefore depends on components that direct their recruitment to specific chromatin domains. In a classical model, described in *Drosophila melanogaster*, PRC2 is first recruited on *cis*-regulatory sequences called Polycomb Response Elements (PREs) via consensus motifs for sequence-specific DNA-binding proteins that might interact with PRC2 subunits.^16–18^ PRC2, via its E(z) subunit, the *Drosophila* ortholog of EZH2/1, deposits H3K27me3. This H3K27me3 mark is then recognized by the cPRC1 PC subunit (ortholog of CBX).^7,17,19,20^ Subsequently, Sce — the ortholog of RING1A/B — ubiquitinates H2AK118^10,11^ (Fig. 2a). This model predicts co-occurrence of PRC1 and PRC2 at their target loci.

However, ncPRC1 complexes do not possess CBX subunits that recognize H3K27me3 and only a small subset colocalizes with this PRC2-deposited mark.^15^ Moreover, mammalian ncPRC1s can act upstream of PRC2 by directly recognizing non-methylated DNA in CGIs leading to the ubiquitination of H2AK119 which is then recognized by PRC2-JARID2.^21–24^ These data suggest that ncPRC1 recruitment to a subset of their targets can act upstream of PRC2 recruitment (Fig. 2a).

The existence of mammalian PREs is still controversial.^25,26^ The analysis of PRC2 genome binding identified the enrichment for CGIs characterized by low levels of DNA methylation, that could therefore act as PREs in mammals^27–29^ (Fig. 2a). Thanks to their Polycomb-like (PCL) extended domain, the PCL proteins PHF1, MTF2 or PHF19 preferentially bind unmethylated CpG-containing DNA sequences,^30^ promote PRC2 binding to CGIs^31,32^ and stabilize the dimerization of PRC2.^33^ The accessory subunits — JARID2 and AEBP2 — are also important for PRC2 recruitment, via recognition of the H2AK119ub mark, as well as for deposition of H3K27me3 at specific PcG targets^34^ (Fig. 2a).

Additional mechanisms involve a PRC1-independent transcriptional repression. Indeed, the proteins Bromo Adjacent Homology Domain Containing protein 1 (BAHD1) and BAH Domain And Coiled-Coil Containing 1 (BAHCC1/BAHD2) possess a C-terminal Bromo Adjacent Homology (BAH) domain which recognizes H3K27me3.^35–38^ Moreover, BAHD1 acts as a scaffold protein that recruits additional co-repressors such as Histone DeAcetylases (HDACs).^35,39^ Alternative recruiting mechanisms also involve long non-coding RNAs (lncRNAs) as well as specific transcription factors (reviewed respectively in^40,41^) (Fig. 2a).

Polycomb recruitment is also modulated by the chromatin landscape. Indeed, Trithorax Group (TrxG) proteins counteract Polycomb-mediated gene silencing by decorating chromatin with active histone marks such as H3K4me1/2/3^6^ and a fine-tuned balance between these two complexes is critically important. SWI/SNF and COMPASS complex subunits are the main TrxG proteins, respectively involved in chromatin remodeling and H3K4 methylation.^42^ Interestingly, MLL2/COMPASS binds specific promoters and trimethylates H3K4 to promote MLL2-dependent gene transcription.^43^ Upon loss of MLL2, H3K27me3 decorates MLL2-dependent genes and represses them.^43^ However, in MLL2 depleting context, H3K27me3 spreading is prevented by DNA methylation at CpG islands.^44^ Dual deletion of MLL2 and DNA methylation increases the repressive mark spreading while diluting its level, which ultimately leads to transcription of the corresponding genes.^43^ Moreover, spreading of the PRC2 mark is also counteracted by H3K36me2, which is deposited by NSD1.^45^

In summary, the molecular mechanisms deployed by PcG complexes to specifically target the genome remain a major area of interest with important consequences for understanding how target genes are specified. Coordinating Polycomb action with key developmental orchestrators, including transcription factors, involves a wide spectrum of tissue- and time-specific players. Future studies should provide insight into this complex Polycomb recruitment network.

## PcG protein function in gene silencing

PcG-mediated transcriptional regulation has been widely portrayed as gene silencing and suggested to be mediated by various mechanisms. First, PcG complexes can mediate chromatin compaction^46–48^ (Fig. 2b). In *Drosophila melanogaster*, mutations in cPRC1 genes were shown to induce decompaction of the Hox clusters, followed by ectopic Hox gene expression which began a few hours later.^49^ In *Ring1b*-knockout mouse embryonic stem cells (mESCs) chromatin decompaction and expression of Hox genes occur even though the H3K27me3 repressive mark is still present.^50^ Surprisingly, this phenotype is rescued by a catalytic mutant form of RING1B, suggesting that its E3-ubiquitin ligase is dispensable for PcG-mediated silencing.^50^ This latter result contrasts with research suggesting a role for the H2AK119ub mark in maintaining PcG-dependent repression.^11,24,51,52^

Second, a switch from a transcriptional repressive state to an active state can be induced by competition between BAF — an ATP-dependent chromatin remodeling complex part of SWI/SNF family — and Polycomb complexes.^12^ BAF-dependent eviction of PcG proteins opens chromatin architecture after H3K27me3 and H2AK119Ub depletion.^53^ Strikingly, in a dominant-negative BAF mutant background, accumulation of PRC1 and PRC2 on chromatin does not necessarily trigger changes in chromatin landscapes, suggesting that DNA-accessibility to BAF is PcG-independent.^54^

Third, the maintenance of the repressed state of PcG target genes also depends on the PcG ability to block initiation and elongation of transcription (Fig. 2c). In particular, RING1-mediated ubiquitination maintains RNA polymerase in a poised state.^52,55,56^ In mESCs, PRC2 can methylate Elongin A to block transcription.^57^ The H2AK119ub and H3K27me3 repressive histone marks respectively repress deposition of the H3K4me2/3 and H3K27Ac active histone marks.^58,59^ Moreover, in flies, PRC1-PC binds to CBP and inhibits its H3K27 acetyltransferase activity, contributing to a repressive state^60^ while on the other hand TRX or TRX-related (TRR) association to CBP antagonizes PcG-mediated silencing.^61^ Interestingly, while the trimethylated form of H3K27 has been extensively studied, less is known about the importance of H3K27me2 in transcriptional repression. Remarkably, the dimethylated H3K27 mark represents 70% of total histone H3 against 4% for the trimethylated form.^62^ Their distribution is mutually exclusive, indeed, the methylated state of H3K27 correlates with different transcriptional states. It is suggested that H3K27me2 coats most of chromatin in order to protect chromatin changes mediated by Histone Acetyl Transferase (HAT).^62^

Finally, PcG proteins actively participate in the three-dimensional (3D) organization of the genome, adding a higher-order layer through which they contribute to gene regulation (Fig. 2d). PcG proteins can drive the formation of 3D-loops between regulatory elements such as promoters and enhancers.^63,64^ Loop formation involves the cPRC1-PH subunit, that oligomerizes via its SAM domain, but is independent of the cPRC1 catalytic activity.^65–67^ Consistent with a function for 3D architecture in gene regulation, PRC1 knockout in ESCs leads to loss of promoter–promoter contacts resulting in transcriptional upregulation of PRC1 target genes.^68^

## PcG protein function in transcriptional activation

Interestingly, an involvement of PcG proteins in transcriptional activation has been suggested in pathological as well as in physiological contexts^69^ and is now better understood at the molecular level (reviewed in^70^). Morey et al. described that only 31% of the cPRC1 and ncPRC1 target genes overlap.^71^ While cPRC1 target genes are strongly repressed, ncPRC1 target genes are overall expressed and involved in dynamic processes such as metabolism and cell cycle progression.^71^ Thus, H3K27me3-independent PRC1 recruitment appears to be an important feature that favors transcriptionally active states by PcG proteins.^72–74^ For instance, PRC1.5 includes the component Autism Susceptibility candidate 2 (AUTS2) that recruits CK2 and p300 which, respectively, inhibits the E3-ubiquitin ligase activity by phosphorylating RING1B and deposits acetylation on histone tails to facilitate transcription.^15,75^ For PRC1.5-AUTS2 target genes, the concomitant enrichment for the H3K4me3 and H4K16Ac active marks, the presence of the RNA polymerase II and a reduction of the H3K27me3 repressive mark lead to transcriptional activation.^75^ The transcription factor NRF1 is involved in PRC1.5-AUTS2 recruitment to its target genes, providing an example for sequence-specific targeting of a PRC1 complex in mammals.^76^

To recapitulate, it is the specific composition of the PcG complexes, as well as the dynamics of their chromatin binding and replacement through cell lineages that determine their transcriptional impact.^15,71,77^ While canonical PcG proteins maintain cellular memory, such as in stem cells where they support self-renewal properties by repressing lineage-specific genes, ncPRC1s control differentiation in more subtle ways. By fine-tuning transcription, PcG proteins are master contributors of cell fate determination,^1^ in particular in the control of a balance between proliferation and differentiation. On the other hand, loss of this fine balance upon misregulation of PcG-dependent mechanisms can cause pathogenesis.

## Polycomb in cancer

Altering the proper functions of PcG can affect cellular identity, therefore promoting tumorigenesis (Tables 1, 2).

**Table 1:** Alterations of PRC2 components and their mechanistic consequences in cancer.

Subunit	Alterations			Functions	Cancer type	Ref
EZH2	Gain-of-function mutations: Y641F/N/S/H/C, A677G/V, A687V Overexpression			Hypermethylation of PcG target genes, increase protein stability	DLBCL, follicular lymphoma, MSD, prostate cancer	^80–82,99–102^
	Loss-of-function mutations, deletions			Hypomethylation of PcG target genes leading to expression of oncogenic genes such as Notch	T-ALL	^108^
	Overexpression				Hematological malignancies, pancreatic cancer, prostate cancer, breast cancer	^232^
	PTMs	Acetylation	K348	Oncogenic function, PCAF-mediated acetylation decreases T345 and T487 EZH2 phosphorylation and increases protein stability, enhancing EZH2 ability to repress transcription and promote cell migration and invasion in lung cancer	Lung cancer	^233^
		Deubiquitination	K222	Oncogenic function, MELK phosphorylates S220 of EZH2 which recruits USP36 to deubiquitinate K222 and stabilize EZH2	NKTL	^234^
		Methylation	K307	Oncogenic function, SMYD2-mediated methylation enhances protein stability	Breast cancer	^112^
			R342	Oncogenic function, PRMT1-mediated methylation inhibits EZH2-T345/T487 CDK1-mediated phosphorylation which increases EZH2 stability. EZH2 stability increases breast cancer metastasis	Breast cancer	^110,235^
			K735	Tumor suppressive role by decreasing protein stability and reducing prostate cancer metastases	Prostate cancer	^111^
		O-GlcNAcylation	S73	Enhance EZH2 protein stability and its catalytic activity, participating in tumor progression	Breast cancer	^236,237^
			S76			
			S84			
			S87			
			T313			
			S729			
		Phosphorylation	S21	Akt-mediated phosphorylation suppresses EZH2 catalytic activity due to its inability to bind H3, resulting in derepression of PcG target genes	Breast cancer, GBM	^238,239^
			S220	MELK-mediated phosphorylation promotes USP36-mediated deubiquitination of K222	NKTL	^234,240^
			Y244	JAK3-mediated phosphorylation inhibits the formation of PRC2, EZH2 then associates with RNA Polymerase II leading to transcriptional activation		
			T261	CDK5-mediated phosphorylation is required for FBW7-mediated EZH2 degradation which inhibits tumor progression	Pancreatic cancer	^241^
			T311	Tumor suppressive function, AMPK-mediated phosphorylation disrupts the EZH2 and SUZ12 association which decreases PRC2-mediated silencing up-regulating tumor suppressor genes	Breast cancer, ovarian cancer	^242^
			T350	CDK1-mediated phosphorylation enhances the ability of EZH2 to bind HOTAIR lncRNA	Lung cancer, breast cancer	^110,233,243,244^
			T492	Tumor suppressive function, CDK1-mediated phosphorylation inhibits the methyltransferase activity of EZH2 by disrupting its binding to core PRC2 proteins	Breast cancer, lung cancer	^110,233,245^
			S363	Tumor suppressive function, GSK3β-mediated phosphorylation reduces EZH2-mediated silencing	Breast cancer	^246^
			T367	Oncogenic function, p38-mediated phosphorylation induces an EZH2 cytoplasmic localization which participates in the process of metastasis	Breast cancer	^247^
			T416	Oncogenic function, CDK2-mediated phosphorylation promotes tumorigenesis	Breast cancer	^248,249^
			Y641	Tumor suppressive function, JAK2-mediated phosphorylation with β-TrCP-mediated ubiquitination leads to EZH2 degradation and hypomethylation of PcG target genes.	Lymphoma	^250^
		Ubiquitination	K421	Tumor suppressive function, Smurf2-mediated ubiquitination leads to EZH2 degradation		^251^
SUZ12	Loss-of-function mutations, deletions			Hypomethylation of PcG target genes, leading to oncogenic-related expression of Notch pathway genes	T-ALL	^108^
	Overexpression			Oncogenic function by repressing tumor suppressor genes (e.g., HRK), and promoting oncogene expression (e.g., cyclin D1)	Ovarian, colorectal, HNSCC	^113–115^
	Downregulation			Decreased expression of Suz12 leads to increased activation of the ERK1/2 pathway and increases expression of MMP9 and MMP2 which promotes migration and invasion	Hepatocellular carcinoma	^252^
EED	Loss-of-functions mutations: I363M			Impaired EED binding to H3K27me3, decrease in PRC2 catalytic activity	Myelodysplastic syndrome	^90,253^
	Overexpression			Promotes EMT by silencing E-cadherin	Breast cancer, colorectal cancer, hepatocellular carcinoma	^232,254^
JARID2	Overexpression			Oncogenic function by increasing the deposition of the repressive mark at the PTEN promoter which promotes invasion and metastasis	Ovarian cancer cell lines, rhabdomyosarcomas, hepatocellular carcinoma	^255–257^
	Deletion			JARID2 plays a tumor suppressive function by repressing self-renewal pathways	Chronic myeloid disorders	^258^
AEBP2	Overexpression increase protein stability			Oncogenic function and chemoresistance	Ovarian cancer	^259^
EPOP	Overexpression			Oncogenic function that may depend on its interaction with Elongin BC and USP7 in order to modulate the chromatin landscape	Breast cancer, colon cancer	^260^
PCL1/PHF1	Fusion genes: JAZF1-PHF1, EPC1-PHF1, BRD8-PHF1, MEAF6-PHF1			Might have an oncogenic function by deregulating the gene expression of target genes by altering the chromatin accessibility	Endometrial stromal sarcoma, ossifying fibromyxoid tumor	^261–263^
PCL2/MTF2	Overexpression			Oncogenic function, upregulates EZH2 and EED expression levels and the level of H3K27me3, H3K4me2 and H3K9me2	Gliomas	^264^
				Tumor suppressive function, inhibits cell proliferation and promotion of apoptosis by inhibiting MDM2-mediated p53 degradation	Breast cancer	^265^
PCL3/PHF19	Overexpression			Tumor suppressive function, inhibits invasion and angiogenesis by interacting with PRC2	Prostate cancer, melanoma	^266,267^
	Overexpression, genomic amplification			Oncogenic function, increases PRC2 activity	Hepatocellular carcinoma, glioblastoma cells, multiple myeloma	^268–270^
CATACOMB	Fusion genes: MBTD1-CXorf67			Recurrent fusion of unknown mechanistic function	Endometrial stromal sarcoma	^209^
	Overexpression			Oncogenic function, mimics H3K27M oncohistones by binding the SET domain of EZH2 which blocks its catalytic activity leading to the derepression of PRC2 targeted genes	PFA	^9,271^

DLBCL, Diffuse Large B-Cell lymphoma; GBM, Glioblastoma; HNSCC, Head and Neck Squamous Cell Carcinoma; NKTL, Natural Killer/T-cell Lymphoma; PFA, Posterior Fossa A; T-ALL, T-cell Acute Lymphoblastic Leukemia.

**Table 2:** Alterations of PRC1 components and their mechanistic consequences in cancer.

Subunit	Alterations			Functions	Cancer type	Ref
RING1B	PTMs	Phosphorylation	S41	Oncogenic function, promotes the recruitment of demethylase UTX and acetylase p300 at poised promoters (e.g., *CCDN2*)	Melanoma	^272^
	Overexpression			Oncogenic function by enhancing oncogene expression (e.g., p63)	AML, breast cancer, gastrointestinal tumors, lymphomas, pancreatic cancers	^138,273,274^
				Oncogenic function by regulating oncogenic enhancer activity via its association with pioneer factors (e.g., FOXA1) and transcription factors (e.g., ERα)	Breast cancer, leukemia, hepatocellular carcinoma	^139^
RING1A	Overexpression			Oncogenic function by enhancing oncogene expression	AML	^138^
PCGF1	Overexpression			Oncogenic function through repression of p21^Waf1/Cip1^	HeLa cells	^275^
				Oncogenic function by promoting cancer stem cell self-renewal via the direct repression of RDH16, leading to the decrease in the synthesis of all-trans retinoic acid	Glioma cells	^276^
				Oncogenic function by enhancing the expression of CRC stem cell markers (e.g., CD133, CD44, ALDH1A1) via an increase in H3K4me3, while H3K27me3 decreases following upregulation of KMT2A and KDM6A	Colorectal cancer	^277^
PCGF2/MEL-18	Overexpression			Tumor suppressive function by inhibiting the expression of oncogenes (e.g., PI3K/AKT pathway, ZEB1/ZEB2, PCGF4)	Breast cancer, gastric cancer	^132,134,278,279^
PCGF3	Overexpression			Oncogenic function by promoting proliferation via expression of cell cycle-related genes (e.g., *CyclinB1*, *CyclinD1*, *CDK4*), migration-related genes (*RhoA*, *RhoC*, *CDC42*) and by regulation of the PI3K/AKT pathway	NSCLC	^280^
PCGF4/ BMI-1	Overexpression			Oncogenic function, promotes cell immortality by repressing the *INK4a-ARF* locus	Breast cancer, NSCLC, gastric carcinoma, pancreatic cancer, hematological malignancies	^124,281–284^
	IG-BMI1 fusion			Oncogenic function	Chronic lymphocytic leukemia	^285^
	Enhanced protein stability			Its association with AR inhibits its degradation, leading to the overexpression of AR downstream target genes (PSA, TMPRSS2)	Prostate cancer	^163^
	PTMs	O-GlcNAcylation	S255	O-GlcNAcylation increases BMI-1 protein stability, which in turn represses TP53, PTEN and CDKN1A/CDKN2A	Prostate cancer	^162^
PCGF6	Frameshift deletion inducing an early stop of translation			Oncogenic function, promotes cell migration by affecting the EMT pathway	Breast cancer	^286^
CBX1	Overexpression			Oncogenic function	Hepatocellular carcinoma, breast cancer	^287,288^
CBX2	Overexpression			Oncogenic function by activation of the PI3K/AKT pathway	Breast cancer	^289^
				Oncogenic function by activation of the YAP/β-catenin pathway	Gastric cancer	^290^
				Oncogenic function by inducing chemoresistance, stem cell-like phenotype	High-grade serous ovarian carcinoma	^291^
CBX3	Overexpression			Oncogenic function via transcriptional regulation of p21 leading to excessive proliferation	Colon cancer, tongue squamous cell carcinoma	^292,293^
				Oncogenic function, higher expression correlates with poor prognosis	Breast cancer, NSCLC	^294,295^
				Oncogenic function via FBP1 silencing which positively regulates aerobic glycolysis	Pancreatic cancer	^296^
CBX4	Overexpression			Oncogenic function by increasing angiogenesis via the sumoylation of HIF-1α which enhances VEGF expression	Hepatocellular carcinoma	^297,298^
				Oncogenic function via interaction with HDAC1 which transcriptionally represses the tumor suppressor KLF6	Clear cell renal cell carcinoma	^299^
				Tumor suppressive function by repressing Runx2 expression via recruitment of HDAC3 at its promoter, leading to the inhibition of cell migration, invasion and metastasis	Colorectal carcinoma	^300^
CBX6	Overexpression			Oncogenic function by accelerating EMT process in HCC cells via upregulation of Snail and Zeb1	Hepatocellular carcinoma	^301,302^
	Downregulation			CBX6 plays a potential tumor suppressor function by down-regulating BST2. In breast cancer, CBX6 expression is repressed in a PRC2-dependent manner	Breast cancer	^303^
CBX7	Overexpression			Oncogenic function via inhibition of the *INK4a-ARF* locus	Prostate cancer, germinal center-derived follicular lymphomas, gastric cancer	^304–306^
	Downregulation			CBX7 plays a tumor suppressive function. Progressive loss of CBX7 expression as INK4a-ARF expression increases	Thyroid cancer	^307^
				CBX7 plays a tumor suppressive function through inhibition of cyclin E expression	Lung cancer	^308^
				CBX7 plays an oncogenic function via loss of tumor suppressor miRNAs (*miR-125b*) and gain of oncogenic miRNAs (*miR-182* and *miR-183*)	Breast cancer	^309^
CBX8	Overexpression			Oncogenic function	Glioblastoma, breast cancer	^310,311^
				Oncogenic function, binds EGR1 promoter and miR-365-3p, which enhance the AKT/β-catenin pathway	Hepatocellular carcinoma	^312^
				In a PRC1-independent manner, cooperates with Wdr5 in order to maintain H3K4me3 at the level of the promoters of Notch pathway genes	Breast cancer	^313^
PHC3				Tumor suppressive function	Osteosarcoma	^314,315^
	G201C missense mutation			Loses tumor suppressive function		
RYBP	Cytoplasm-located mutant of RYBP			Tumor suppressive function, more potent ability to bind to caspase 8 which prevents p53 degradation in human tumor cells	Breast cancer cells, osteosarcoma epithelial cells, colon cancer cells	^316^
	Overexpression			Oncogenic function	Oligodendroglia tumors, pituitary adenoma, Hodgkin’s lymphoma and T cell lymphoma	^317,318^
	Downregulation			RYBP plays a tumor suppressive function by decreasing proliferation, cell migration and metastasis	Lung cancer, hepatocellular carcinoma, thyroid cancer, breast cancer	^317,319,320^
YAF2	Overexpression				NSCLC	^321^
	PTMs	Phosphorylation	S167	Stabilizes FANK1 and inhibits its degradation, inhibits FANK1-mediated apoptosis	Breast cancer cells, colon cancer cells	^322^
AUTS2	Overexpression			The association of AUTS2 with PRC1.5 favors MSX1 expression, which inhibits the differentiation of T-cell lymphocytes	T-ALL	^323^
	Fusion genes: PAX5-AUTS2			Recurrent rearrangement between 9p13 and 7q11 regions in B-cell acute lymphoblastic leukemias. Mechanism of action unknown to date.	B-ALL	^324^
BCOR	Fusion gene: ZC3H7B-BCOR, BCOR–MAML3			Dysregulation of PRC1-mediated repression which could explain tumorigenesis	Endometrial stromal sarcomas, clear cell sarcoma of the kidney	^325,326^
	Loss-of-function			BCOR plays a tumor suppressive function by inhibiting proliferation and self-renewal mediated by Notch1 target genes	AML, T-ALL	^327^
KDM2B				Tumor suppressive function by suppressing genes of the Notch pathway	T-ALL	^328^
	Overexpression			Oncogenic function, by regulating cell fate	AML, Breast cancer	^329,330^

AML, Acute Myeloid Leukemia; AR, Androgen Receptor; B-ALL, B-cell Acute Lymphoblastic Leukemia; CRC, Colorectal Cancer; ER, Estrogen Receptor; EMT, Epithelial-Mesenchymal Transition; NSCLC, Non-Small Cell Lung Cancer; PTMs, Post-Translational Modifications; T-ALL, T-cell Acute Lymphoblastic Leukemia.

### PRC2 in cancer

**Fig. 3:**
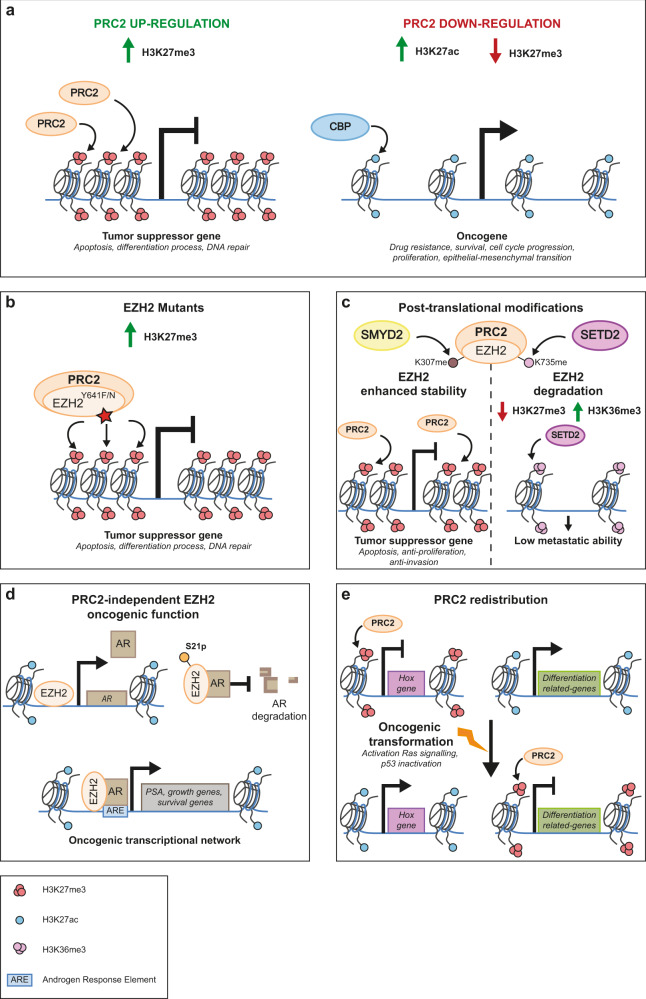
Multifaceted roles of PRC2 in tumorigenesis. **a** Upregulation of PRC2 components results in H3K27 hypermethylation, which, if present in tumor suppressor genes, induces their downregulation. In contrast, downregulation of PRC2 components at oncogenes leads to H3K27 hypomethylation and a switch to acetylation, contributing to the overexpression of specific oncogenes. **b** GOF mutations (indicated by a star) affecting the SET-domain of EZH2 can lead to overactivation of its H3K27 methyltransferase catalytic activity and to the silencing of tumor suppressor genes. **c** PTMs of EZH2 participate in tumorigenesis. Left: methylation of K307 of EZH2 by SMYD2 enhances its stability, resulting in a H3K27 hypermethylated state of tumor suppressor genes. Right*:* on the other hand, methylation of its K735 causes EZH2 degradation. The loss of EZH2 induces the replacement of H3K27me3 by H3K27ac, leading to the transcriptional expression of oncogenes. **d** Polycomb-independent roles of EZH2 in transcriptional activation. The gene encoding the AR is a direct target of EZH2-mediated transcriptional activation in Androgen-Dependent and Castration-Resistant Prostate Cancers (ADPC and CRPC, respectively). This mechanism is methylation-independent and escapes EZH2 inhibitors. In CRPC, EZH2 acts as a co-factor of AR. This functional transition of EZH2 from a role of repression to a role of activation of transcription depends on its phosphorylation at the level of Ser21. EZH2 and AR directly interact. This interaction inhibits the degradation of the AR and causes the overexpression of the AR target genes. **e** Under physiological conditions, PRC2 participates in the transcriptional repression of its HOX target genes throughout development. However, oncogenic transformation can redirect PRC2 to new target genes. This PRC2 redistribution, in particular at differentiation-related genes, induces a loss of differentiation and participates in the generation of a pluripotent stem cell-like phenotype. AR, Androgen Receptor; CBP, CREB binding protein; PSA, Prostate-Specific Antigen; SETD2, SET domain-containing 2 (a histone lysine methyltransferase); SMYD2, SET and MYND domain-containing 2.

Polycomb dysregulation in cancer has been the subject of extensive studies since Varambally et al. demonstrated that EZH2 overexpression is associated with advanced stage and poor prognosis in prostate cancer.^78^ Quantitative and qualitative EZH2 dysregulation has been frequently described in solid malignancies including lung, hepatocellular, breast, colorectal, pancreatic cancers as well as in several hematologic malignancies.^79,80^ *EZH2* expression can be regulated by specific transcription factors, including the MLL-AF9 fusion protein, or by miRNAs that will induce *EZH2* mRNA decay.^81,82^ Dysregulation of those specific regulators participates in the tumorigenesis onset. *EZH2* overexpression in patients is associated with a higher risk of relapse.^78^ PRC2 plays a major role in self-renewal of hematopoietic stem cells;^83–86^ its dysregulation is often found in multiple blood cancers^87–92^ in which EZH2 can behave both as a tumor suppressor^85,93,94^ or an oncogene^95–98^ depending on the cell context (reviewed in^80^) (Fig. 3a).

The onset or cancer progression may be associated with mutations affecting the catalytic SET-domain of EZH2 that is essential for H3K27 methylation (Fig. 3b). An EZH2^Y641F/N^ gain-of-function (GOF) mutation affecting the tyrosine 641 (Y641) located in the SET-domain induces hypermethylation of H3K27.^99,100^ Particularly, EZH2^Y641^ has an increased affinity for dimethylated H3K27 form which causes a widespread redistribution of H3K27me3 and a decrease in H3K27me2, leading to transcriptional misregulation of affected genes.^100–102^ Moreover, the higher-order chromatin landscape can also be affected. In recent years, multiple cutting edge approaches have shown that the genome folds into a hierarchy of structures, from nucleosomes, to chromatin loops and nanodomains, Topologically Associating Domain (TADs), chromosome compartments and chromosome territories.^103^ TADs are particularly interesting since they constitute regulatory landscapes for the genes contained within each TAD.^104^ Interestingly, co-repression of several tumor suppressors was suggested to participate in tumor growth.^105,106^ An established tumor state can also participate *a posteriori* in the redistribution of EZH2 on ectopic targets, triggering changes in cell identity due to misexpression of homeotic genes.^107^ In addition to GOF effects, loss-of-function (LOF) mutations and deletions affecting *EZH2* and *SUZ12* in T-cell acute lymphoblastic leukemia (T-ALL) — a hematopoietic cancer — lead to hypomethylation of H3K27 target genes, including *Notch*, a major player in T-ALL, thereby contributing to oncogenesis^108^ (Fig. 3a). PRC2 LOF is found in around 25% of T-ALL in association with oncogenic activating mutations of the JAK/STAT signaling pathway and leads to a global epigenetic remodeling towards H3K27Ac. This active histone mark is recognized by Bromodomain and Extraterminal (BET)-domain proteins that act as its specific readers, allowing reactivation of a BET-dependent transcriptional network that triggers stem cell-like programs leading to poor prognosis. PRC2-altered T-ALL being dependent on BET proteins, BET domain protein inhibition is therefore a promising therapeutic avenue in PRC2-associated-T-ALL patients.^109^

EZH2 post-translational modifications (PTMs) play an additional role in certain type of cancers^110,111^ (Fig. 3c). In patients with advanced prostate cancer, H3K36me3 and H3K27me3 levels are inversely correlated.^78,111^ SETD2, the methyltransferase responsible for H3K36me3 deposition, also monomethylates EZH2 on its lysine 735 residue, inducing EZH2 degradation and consequently delaying metastasis. SETD2 is strongly correlated with the presence of EZH2-K735me1 and particularly found in patients with prostate cancer with better clinical outcome.^111^ On the contrary, EHZ2-K307 methylation by SMYD2 improves its stability and participates in the transcriptional repression of pro-apoptotic, anti-proliferation and anti-invasion target genes^112^ (Fig. 3c). Multiple EZH2 PTMs play thus a role in EZH2 function and stability that will result in an H3K27 hypermethylation or hypomethylation of the chromatin landscape that favors tumorigenesis (Table 1).

Additionally, SUZ12 is upregulated in a variety of cancers, including ovarian, colorectal and head and neck squamous cell carcinoma.^113–115^ The knockdown of *SUZ12* is able to reverse tumor growth by inhibiting proliferation and inducing apoptosis in these contexts.^113,115^ On the other hand, SUZ12 loss in T-ALL disrupts the PRC2 complex, leading to H3K27me3 decrease which correlates with the opening of chromatin and upregulation of the corresponding genes involved in oncogenic signaling pathways^92^ (Fig. 3a). Moreover, PRC2 loss induces a genome-wide redistribution of the H3K27Ac mark and the activation of poised enhancers.^62^ Therefore, similar to EZH2, SUZ12 can act as pro-oncogenic or tumor suppressor depending on the cancer type.

As previously mentioned, PRC2 can be divided into two sub-complexes, PRC2.1 and PRC2.2. While their target genes are overlapping,^34,116^ their differences rely on their affinity to chromatin.^117^ Indeed, PRC2.1 tends to have a higher affinity to chromatin, which leads to an increase in H3K27me3 deposition and silencing of PcG target genes in the presence of high ratios of PRC2.1 to 2.2.^117^ In leukemia, colon and uterine adenocarcinomas, missense mutations of SUZ12, SUZ12(R103P/Q), result in JARID2 depletion, leading to an increase in PRC2.1 formation which enhances PRC2 chromatin occupancy.^117^ How PRC2.1 could be specifically implicated in cancer remains to be determined.

Although PRC2 dysregulation events have been widely documented in cancer, it is still difficult to decipher whether they are drivers in tumorigenesis. Even if *EZH2* is dispensable for the progression of prostate and mammary cancer, it is nonetheless highly expressed.^118^ In fact, in normally dividing cells, the rate of *EZH2* expression correlates with proliferation rates,^118^ compensating the proliferation-dependent dilution of H3K27me3. In these cancers, even though EZH2 is overexpressed, tumor cells paradoxically fail to maintain a wild-type dose of H3K27me3. The use of EZH2 inhibitors for cancer treatment should therefore carefully take into account the tumor proliferation status.^118^ With the aim to identify the cancer types in which treatment using PRC2 inhibitors could be beneficial, a genomic and transcriptomic analysis using available databases on clinical tumor samples and a panel of tumor cell lines has been performed, revealing a correlation of EZH2, SUZ12 or EED amplifications with poor prognosis in a subclass of human cancers like renal papillary cell carcinoma, low-grade glioma and hepatocellular carcinoma.^119^ Interestingly, GOFs of PRC2 subunits are also anti-correlated with poor prognosis in some cancers like gastric cancer and thymoma, suggesting a tumor suppressor function of PRC2 in those cases.

It remains to be understood why certain tumors are addicted to one specific PRC2 subunits but not the others. Clearly, a better understanding of the rate-limiting roles and the cell type-specific functions of each of the PRC2 subunits will require future research.

### PRC1 in cancer

**Fig. 4:**
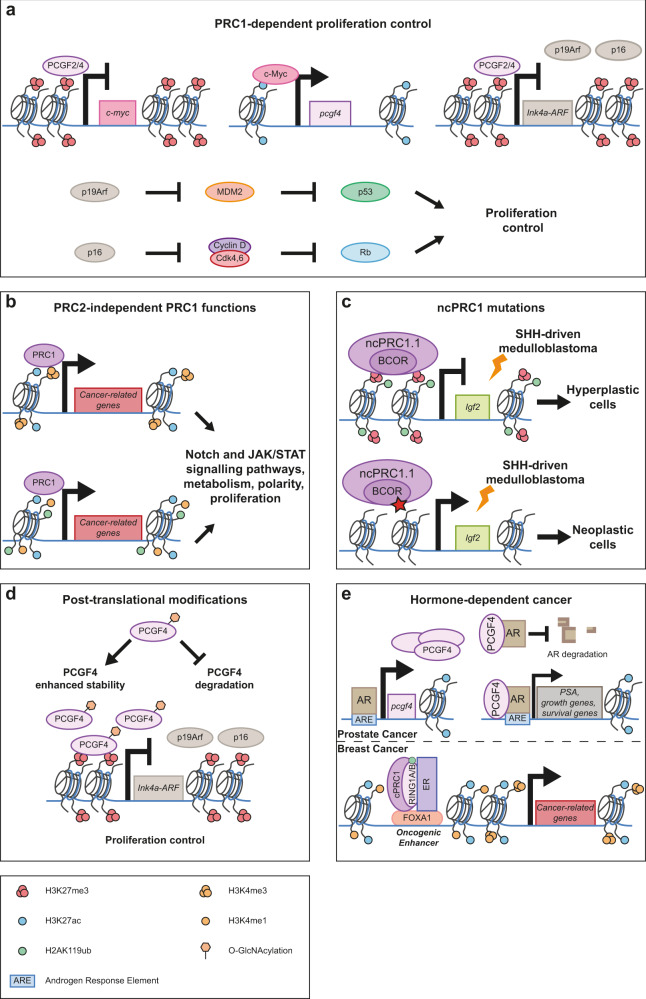
Multifaceted roles of PRC1 in tumorigenesis. **a** PCGF2 inhibits the transcription of *c-myc*. Loss of c-Myc results in the decrease of PCGF4 expression, and in the derepression of PCG4 target genes, such as the *INK4a-ARF* locus. p19 and p16 participate in proliferation control, respectively, by inhibiting MDM2-mediated degradation of p53 and inhibiting CycD/CDK4-mediated phosphorylation of pRb. **b** PRC1 oncogenic activity may also be PRC2-independent. PRC1 is found on specific targets lacking the H3K27me3 repressive mark. Surprisingly, these genes exhibit active marks such as H3K27Ac and H3K4me1/3. Gene ontology analysis characterized these cancer-related genes as components of cell signaling, like the Notch and JAK/STAT signaling pathways. **c** PRC1 mutations are rarely found in cancer, although some mutations have been found to impact variant PRC1. Indeed, mutations (indicated by a star) in BCOR, a scaffold protein involved in ncPRC1.1, are found in SHH-driven medulloblastoma. The presence of these mutations promotes a neoplastic state of cancer cells by preventing Polycomb recruitment to its target genes. **d** PTM of PRC1 subunits can promote tumorigenesis. The deposition of O-GlcNAcylation on PCGF4 (BMI-1) inhibits its degradation. PCGF4 protein levels are increased and participate in the transcriptional silencing of downstream target genes such as the *INK4a*-*ARF* locus, thus promoting oncogenic cell proliferation. **e** In hormone-dependent cancers, PRC1 genes are often amplified. Top: in prostate cancer, the AR promotes the expression of PCGF4. Additionally, it can interact with the PCGF4 protein, resulting in inhibition of AR degradation and transcriptional activation of its downstream target genes. Bottom: cPRC1 can also interact with the ER and its pioneer factor FOXA1 in ER^+^ breast cancer cells and bind to enhancers that stimulate transcription of cancer-related genes decorated with active histone marks. AR, Androgen Receptor; Cdk4, 6, Cyclin Dependent Kinase 4, 6; ER, Estrogen Receptor; FOXA1, Forkhead Box A1; Igf2, Insulin-like growth factor 2; MDM2, Murine Double Minute 2; PSA, Prostate Specific Antigen; Rb, Retinoblastoma.

Like PRC2, PRC1 components are widely implicated in many types of cancers (Table 2). BMI-1 (PCGF4), a cPRC1.4 subunit, has historically been described as a proto-oncogene that collaborates with the c-Myc oncoprotein to trigger tumorigenesis.^120–123^ The *INK4a-ARF* locus, encoding the tumor suppressors p16^Ink4^ and p19^Arf^, is a direct target of PRC1.4.^124^ BMI-1 deficiency is associated with overexpression of p16 ^Ink4^ and p19^Arf^ and therefore with cell cycle arrest, senescence and apoptosis (Fig. 4a). In contrast, BMI-1 overexpression triggers cell proliferation by repressing *ink4a-ARF* expression.^124^ BMI-1 is involved in gastric, pancreatic, breast and ovarian cancer among others.^125–129^ MEL-18 (PCGF2), a BMI-1 homolog, has a tumor suppressing activity.^130–132^ BMI-1 and MEL-18 expression levels are inversely correlated in various cancers.^133,134^ BMI-1 expression depends on its counterpart MEL-18 (Fig. 4a). c-Myc is a transcriptional activator of BMI-1. *Mel-18* overexpression is linked to *c-myc* downregulation, leading to BMI-1 decrease, p16 upregulation and ultimately to cell senescence.^135^ Interestingly, in flies, LOF of cPRC1 members results in upregulation of cancer-related genes, including genes involved in the Notch, JNK and JAK/STAT signaling pathways^74,136,137^ (Fig. 4b), a difference that might be due in part to the absence of PcG-mediated repression of the *INK4a-ARF* locus in flies.

Using an in vivo and in vitro approach, ncPRC1.1 was shown to specifically target active genes independently of PRC2.^74,138^ At a genome-wide level, the correlation between mammalian RING1B and the H3K27me3 mark decreases during lineage decision processes. While PRC1-RING1B targets are clearly enriched for the repressive H3K27me3 mark in ESCs, this is only the case for ~30% of them in differentiated cells.^74^ While gene ontology categories associated with H3K27me3-dependent targets are linked to developmental pathways, H3K27me3-independent targets are linked to cell cycle regulation, cell polarity, metabolism and signaling pathways^74,138^ (Fig. 4b). This difference in PRC1 targeting results from major changes in the qualitative and quantitative compositions of the ncPRC1 variant complexes.^15,71^

Unlike PRC2 mutations, PRC1 mutations are not overrepresented in cancer.^139^ However, some mutations affecting ncPRC1 have been described.^140,141^ In SHH-driven medulloblastoma, the PRC1.1 BCOR scaffold protein is mutated at its C-terminal domain that normally interacts with PCGF1,^141,142^ resulting in loss of PRC1.1 recruitment to genes coding for growth factors that would otherwise be repressed^141^ (Fig. 4c). Likewise, MGA, a transcription factor that is a member of the Myc network and interacts with ncPRC1.6 subunits, is a tumor suppressor in vivo that acts by recruiting ncPRC1.6 to its target genes.^143^ Moreover, BAP1, a component of the Polycomb Repressive complex DeUbiquitinase (PR-DUB), is a tumor suppressor.^144,145^ Recent data suggest that this protein prevents widespread H2AK119ub deposition and chromatin condensation at non-target loci, restricting H2AK119ub to Polycomb target genes. BAP1 may thus prevent inappropriate redistribution of Polycomb complexes away from their targets and play critical roles, particularly by maintaining the appropriate chromatin state of lineage commitment genes.^146–149^ It is therefore not surprising that PR-DUB misregulation leads to tumorigenesis. Enhancing deubiquitinase activity leads to a widespread depletion of the H2AK119ub mark.^140^ Conversely, disruption of its chromatin recruitment or catalytic activity could result in an increase in H2AK119ub and H3K27me3.^146,150^ Depending on the genes targeted, this might switch the transcriptional state of oncogenes or tumor suppressor genes.

In summary, the implication of PcG components in cancer, either by point mutations or by dysregulation of its components, is widely established. Through tumor suppressor or oncogenic activity in a broad type of cancers, PcG members control tumor growth and survival.^151^ Targeting PRC2 members or proteins involved in PRC2 stability, either by inhibiting its enzymatic activity or by interfering with PRC2 complex assembly or stability, appears to be a promising strategy to prevent growth of PRC2-dependent tumors^79,152–154^ (Table 3). However, since PRC1 can either repress or activate the transcription of its target genes, it is both the downregulation and/or upregulation of tumor suppressors and oncogenes respectively that might participate in tumorigenicity.^69,155–157^ The exact role of PRC1 complexes in cancer, and in particular the importance of ncPRC1 complexes, remains to be determined. Future work would be important to better characterize the molecular implication of Polycomb complexes and define appropriate therapeutic approaches to rescue their dysregulation in different types of cancer.

**Table 3:** PcG inhibitors and ongoing clinical trials.

Target	Agent	Cancer	Status	Clinical study (NCT#)	Ref
EZH2	Tazemetostat (formerly known as: EPZ-6438, E7438)	B-cell NHL	Phase 2	NCT03456726	
		MRT, RTK, ATRT, synovial sarcoma, malignant rhabdoid tumor of ovary, renal medullary carcinoma, epitheloid sarcoma, solid tumor with an EZH2 GOF mutation	Phase 2	NCT02601950	^331^
		Malignant mesothelioma	Phase 2	NCT02860286	
		B-cell lymphomas, advanced solid tumors DLBCL, follicular lymphoma	Phase 1 Phase 2	NCT01897571	^332,333^
	SHR2554	Lymphoid neoplasm	Phase 1	NCT03603951	
		Solid tumor, lymphoma	Phase 2	NCT04407741	
	CPI-1205	B-cell lymphoma	Phase 1	NCT02395601	^334^
		mCRPC	Phase 2	NCT03480646	
	Valemetostat Tosylate (DS-3201b)	T-cell leukemia/lymphoma	Phase 2	NCT04703192	
		Small cell lung cancer	Phase 1	NCT03879798	
		Lymphoma, non-Hodgkin lymphoma		NCT02732275	

ATRT, Atypical Teratoid Rhabdoid Tumors; DLBCL, Diffuse Large B-cell Lymphoma; GOF, Gain-Of-Function; mCRPC, Metastatic Castration Resistant Prostate Cancer; MRT, Malignant Rhabdoid Tumors; NHL, Non-Hodgkin’s Lymphoma; RTK, Rhabdoid Tumors of the Kidney.

## Environmental cues and polycomb-dependent oncogenesis

### Hormone-dependent cancer

PRC1 genes are significantly amplified in hormone-dependent cancers.^139^ Since hormone receptors are transcription factors, they might participate in tumorigenesis by triggering ectopic recruitment of Polycomb proteins to a specific set of target genes. In particular, the androgen receptor (AR) and the estrogen receptor (ER) can directly recruit PcG proteins at their response elements in hormone-dependent cancers.^139,158–160^ In prostate cancer, maintenance of AR expression is essential. The overexpression of BMI-1 and its increased protein stability mediated by PTMs, such as *O*-GlcNAcylation, participate in the self-renewal of cancer cells and the progression of prostate cancer^161,162^ (Fig. 4d**)**. Furthermore, the binding of BMI-1 to AR inhibits the ubiquitin–proteasome degradation pathway.^163^ Surprisingly, the AR interacts with BMI-1 in a PRC1-independent manner.^163^ By coupling ChIP-seq and CRISPR methodologies, it was found that Androgen Response Elements (AREs) are located in the *BMI1* locus and enriched for the H3K27Ac active enhancer mark, suggesting that the AR activates transcription of BMI-1.^160^ Moreover, a positive feedback loop exists in prostate cancer where BMI-1 overexpression stabilizes AR, which in turn transcriptionally activates BMI-1 expression, leading to tumor progression (Fig. 4e**)**. In addition, a PRC2-independent EZH2 oncogenic function relies on its direct interaction with AR, leading to AR transcription and activation of AR downstream targets^164–166^ (Fig. 3d**)**. This PRC2 genome-wide redistribution also results in ectopic targeting, in particular to tumor suppressor genes, particularly those involved in INF-ɣ signaling, that are repressed by the H3K27me3 mark in prostate cancer^167,168^ (Fig. 3e**)**.

The redistribution of PcG-targets is an important mechanism participating in tumorigenesis and cancer progression. Surprisingly, in breast cancer, ERα, β-catenin and EZH2 interact and target oncogenes, such as *c-Myc* and *Cyclin D1*, acting as transcriptional co-activators.^169^ Furthermore, the redistribution of PRC1 leads to its association with active enhancers enriched for the H3K4me1 mark.^139^ RING1B was proposed to facilitate ERα recruitment to enhancers and super-enhancers, as well as to promoters of cancer-related genes^139,170^ (Fig. 4e**)**. However, how RING1B is recruited to open chromatin sites and how it selectively binds to a subset of them is still unclear.

### Metabolism

**Fig. 5:**
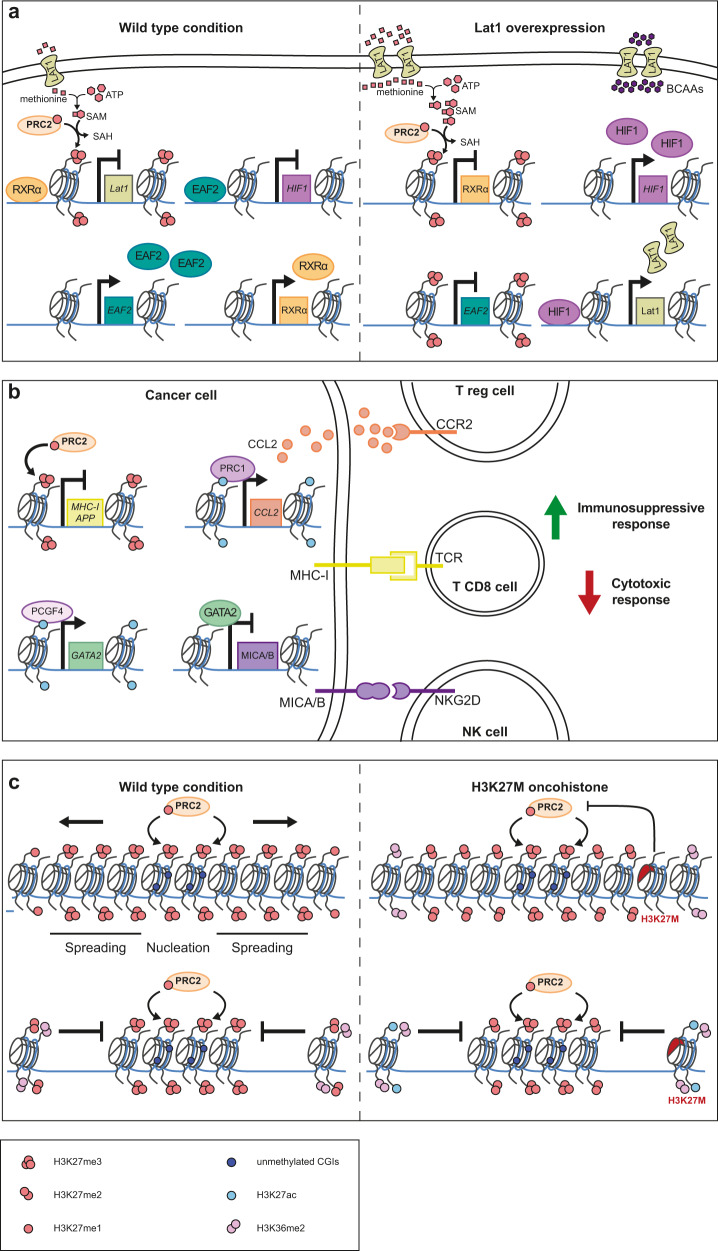
Environment-dependent oncogenic activities of PcG proteins. **a** Left: in a physiological condition, the membrane transporter LAT1 participates in the transport of methionine which reacts with ATP to produce SAM. SAM can in turn be used by PRC2 to induce trimethylation of H3K27, resulting in a PcG-mediated silencing of its targets genes. *Lat1* expression depends on RXRα. Right: in cancer cells, *Lat1* is overexpressed, enhancing SAM production and inducing H3K27 hypermethylation of the chromatin landscape. The *Lat1* negative regulator, RXRα, is thus repressed resulting in a positive feedback loop whereby EAF2 transcriptional silencing dependent on PRC2 results in overexpression of HIF1, which can in turn stimulate *Lat1* expression. Therefore, an excess of LAT1 at the cellular membrane increases the transport of BCAAs, thereby enhancing protein synthesis. **b** Controlling the immune system is of a major importance in cancer. Cancer cells use different mechanisms to do this. First, PRC1 is able to increase the transcriptional expression of CCL2, which will dampen Treg immune response. In addition, PRC2-mediated silencing of the MHC-I antigen processing pathway results in MHC-I absence at the cell membrane, concealing cancer cells from cytotoxic T cells. Finally, PCGF4 overexpression in cancer cells stimulates the expression of GATA2, which will inhibit MICA/B transcription and reduces its presence at the membrane. This prevents the recognition of cancer cells by NK cells. These mechanisms enhance the immunosuppressive response and inhibit the cytotoxic response that would otherwise kill the cancer cells. **c** Oncohistones are a new line of research, analyzing the effect of mutations on histone genes that could have an impact on tumorigenesis. H3K27M has a dominant negative effect on EZH2 catalytic activity. Left: in a wild-type condition, PRC2 is recruited to nucleation sites that present unmethylated CGIs. Trimethylation of H3K27 occurs and spreads around the nucleation site. The boundaries of Polycomb domains are decorated with H3K36me2. Right: in the presence of the H3K27M oncohistone, that represents 10% of all H3, an epigenetic remodeling occurs. The spreading of H3K27me3 is inhibited and active histone marks, such as H3K27ac, are present on the oncohistone. BCAAs, Branched-chain amino acids; CCL2, C-C motif chemokine ligand 2; CCR2, C-C motif chemokine receptor; EAF2, ELL associated factor 2; GATA2, GATA binding protein 2; HIF1, Hypoxia inducible factor 1; IDH1, Isocitrate dehydrogenase 1; KDM6A/B, Lysine demethylase 6A/B; LAT1, L-type amino acid transporter 1; MHC-I, Major histocompatibility complex I; MHC-I APP, Major histocompatibility complex I antigen processing pathway; MICA/B, MHC I polypeptide-related sequence A/B; RXRα, Retinoid X receptor-alpha; SAM, S-adenosylmethionine; SAH, S-adenosylhomocysteine; TCR, T-cell receptor; SETD2, SET domain containing 2 (histone lysine methyltransferase).

Proliferation and growth of cancer cells are known to be associated with an extensive rewiring of metabolism and energy production networks where Polycomb complexes are clearly involved. As already mentioned, changes in methylation of H3K27 participate in tumor progression.^94,108^ Tight regulation of the methyl group available for EZH2 activity is essential to maintain a proper chromatin landscape. The catalytic activity of EZH2 depends on the methyl donor S-adenosylmethionine (SAM)^171^ (Fig. 5e). SAM is formed by the combination of a methionine, which crosses the cell membrane via the LAT1 transporter, and an ATP molecule. Cancer cells with higher levels of LAT1 expression have a more aggressive phenotype.^172^ Upon LAT1 depletion, the SAM pool is significantly reduced, correlating with a decrease in H3K27me3 deposition even if EZH2 protein concentration is constant.^172^ In addition, repression of RXRα, a known negative regulator of LAT1, by the PRC2 complex maintains a positive feedback loop between LAT1 and EZH2, enhancing EZH2 methyltransferase activity^172^ (Fig. 5a). Indeed, EZH2 inhibition via competition with SAM has a potent anti-tumor effect.^173^

PcG proteins are also involved in the regulation of branched-chain amino acids (BCAAs),^174^ key regulatory components for protein synthesis and energy production, both of which are also the fuel of cancer progression.^175^ Enzymes required for BCAA catabolism, known as BCAA aminotransferases (BCATs), are often overexpressed in cancer cells.^176^ In myeloproliferative neoplasms (MPNs), the combination of partial loss of PRC2 and expression of the constitutively active oncoprotein NRAS^G12D^ — a member of the Ras GTPase family — has been shown to lead to BCAT1 expression, which is normally repressed in hematopoietic stem cells.^174^ The increase in BCAT1 results in a larger pool of BCAAs that activates mTOR, a protein kinase known to participate in tumor growth and proliferation.^177^ It should be noted that in patients with Acute Myeloid Leukemia (AML), the expression of *EZH2* and *BCAT1* is inversely correlated, a high expression of BCAT1 being associated with a poor survival outcome.^174^ In glioblastoma cancer cells, rather than modulating BCAT expression, it is the BCAA pool that increases. In this case, EZH2 represses EAF2 which inhibits Hypoxia-Inducible Factor 1 (HIF1).^178^ HIF-1 overexpression participates in the Warburg effect by supporting glycolytic metabolism and upregulating expression of LAT1, the main transporter of BCAAs, which results in an increase in BCAA pool^178,179^ (Fig. 5a, right).

The Warburg effect is the most well-known cancer metabolic alteration, whereby malignant cells use glycolysis rather than oxygen-dependent metabolism. A tight regulation of glucose homeostasis is essential to counter the proliferation of cancer cells. An important node in this pathway is the reaction catalyzed by the enzyme Fructose-1,6-biphosphatase (FBP1). Low FBP1 enzyme activity correlates with higher production of pyruvate, the downstream product of the glycolysis pathway. An over-production of pyruvate corresponds to a greater store of energy available for cancer cell growth. In hepatocellular carcinoma and clear cell renal cell carcinoma, the mRNA levels of *Ezh2* and *FBP1* are inversely correlated due to the presence of the EZH2-dependent H3K27me3 repressive mark at the promoter of the gluconeogenic enzyme-coding gene.^180^ Tumor growth was shown to be thwarted either by a short-hairpin RNA (shRNA) directed against *Ezh2*, or by the reintroduction of FBP1. Interestingly, FBP1 and EZH2 interact directly. In doing so, FBP1 is able to reduce the methyltransferase activity of EZH2 by dissociating the PRC2 complex. This double negative feedback loop provides new insights into the involvement of Polycomb in “oncometabolism”.

Metabolic reprogramming during tumorigenesis is required to better sustain the energy necessary for cancer progression and survival.^181,182^ PcG proteins have been implicated in the regulation of metabolic genes involved in metabolism of fatty acids and pyruvates among others.^139,183^ While the link between PRC2 function and metabolism in physiology and cancer is certain, much work remains to be done in order to understand the molecular underpinnings of this link in different cancer types and to harness them to design effective therapeutic strategies. Noteworthy, with most of the current research focusing on the link between PRC2 and metabolism,^184^ it might also be of interest to examine the involvement of PRC1 in future work.

### Immune system

The immune system has a wide array of cells that protect from foreign bodies, also known as *non-self*. Innate immune cells provide a rapid and nonspecific response while adaptative immune cells have a slower response that relies on a memory process that will be specific to a known foreign object.^185^ In principle, both innate and adaptative immune cells exert an anti-tumor function.

However, cancer cells can develop multiple mechanisms to evade recognition and destruction by the immune system and become resistant to therapy. In prostate cancer, elevated PRC1 levels and activity coincide with epithelial-to-mesenchymal transition (EMT) and stemness signatures. PRC1 directly promotes metastasis at metastatic initiation sites by controlling self-renewal and both cPRC1 and ncPRC1.1 components directly induce transcriptional expression of CCL2 and other pro-metastatic genes that encode cytokines, which suppress the immune response and promote a pro-angiogenic environment^186^ (Fig. 5b). CCL2 expression has an oncogenic function by recruiting immune cells such as M2-type Tumor-Associated Macrophages (TAMs) and T-regulatory cells (Tregs), promoting an immunosuppressive microenvironment favorable to tumor progression. Moreover, Natural Killer (NK) cells are also involved in the innate immune response. Upon recognition of MICA/B by NK cells, an immune cytotoxic response is displayed. However, BMI-1 stimulates GATA2 expression which in turn directly inhibits MICA/B expression (Fig. 5b). Reduction of MICA/B expression on the surface of cancer cells prevents NK cell activation and the cytotoxic response.^187^ The combination of these two escape mechanisms promotes cancer cell progression and metastasis. Pharmacological treatment using a catalytic inhibitor of PRC1 suppresses metastasis by reverting the immunosuppressive microenvironment and promoting the recruitment of NK cells and T effector cells.^186,187^

Cytotoxic T cells (CD8^+^ T) identify cancer cells presenting foreign antigens by their Major Histocompatibility Complex I (MHC-I). An IFN-ɣ response is then induced to kill the cancer cells. In order to survive, cancer cells downregulate the MHC-I antigen processing pathway (MHC-I APP), resulting in decreased presentation of foreign antigens to CD8^+^ T^188^ (Fig. 5b). PRC2 represses transcription of various MHC-I APP components, participating in cancer cell immunosurveillance escape.^189^ Furthermore, PRC2 inhibits anti-tumor immunity by altering the transcriptional landscape of Tregs. Indeed, immunocompetent mice bearing tumors treated with an EZH2 inhibitor show a significant decrease in tumor volume compared to mice deficient in T cells, suggesting an interplay between EZH2 and the T cell immune response.^190^ Tregs promote tumor progression in an EZH2-dependent manner by producing immunosuppressive cytokines and preventing recruitment of T CDC8^+^.^190,191^ Pharmacological EZH2 inhibition induces a change in the production of pro-inflammatory cytokines which promotes anti-tumor activity and significantly increases the ratio between CD8^+^ T and Tregs in the tumor microenvironment.^190^

Cancer immunotherapy has revolutionized the clinical approach in the field of oncology. However, anti-CTLA4, the first monoclonal antibody used in cancer therapy as an immune checkpoint, induces an upregulation of EZH2 expression^191^ which may prevent anti-tumor immunity by inducing an immunosuppressive tumor microenvironment.^190,191^ A synergistic strategy coupling anti-CTLA4 and an EZH2 inhibitor reverses cancer resistance to the immune system.^191,192^ Moreover, considering the involvement of PcG proteins in pluripotency, it is not surprising that PcG proteins are also involved in cancer stem cell (CSC) development and resistance to treatment. Although anti-PD1 immunotherapy is sufficient to recruit CD8^+^ T cells into the tumor microenvironment, it is not sufficient to kill BMI-1^+^ CSCs.^193^ Inhibition of BMI-1 de-represses H2AK119ub-decorated target genes and increases DNA-damage, stimulating the inflammatory response and CD8^+^ T cells recruitment.^193^ In summary, joint targeting of immune checkpoints and PcG proteins appears to be a new promising therapeutic approach to efficiently counter cancer progression by stimulating the immune response.^191–193^

### Oncohistones

As already mentioned, the catalytic activities of “writers” and “erasers” enzymes that modify histone PTMs are often dysregulated in cancer where chromatin landscapes are modified, resulting in aberrant transcription of the corresponding genes.^99,194^ In addition, the lack of recognition of H3K27me3 by the CBX7 “reader” results in a transcriptional de-repression of tumor suppressor genes.^195^ Likewise, the BAHCC1 mutation in its BAH domain leads to upregulation of tumor suppressor genes that dampen tumor progression.^38^

These data point to a direct involvement of histone modifications in tumorigenesis. Indeed, somatic mutations in histone genes occur at high frequency in cancer, and they can exhibit oncogenic properties.^196^ K-to-M/I missense substitutions in histone variants, analyzed from available sequenced genomes of several human cancer types of ~3000 patients, further argue for a driver or contributor effects of the known N-terminal tail mutations affecting H3.^196^ These mutations are particularly frequent in rare malignancies such as glioma and chondroblastoma. This analysis allowed detection of previously unappreciated situations where histones are mutated at low frequency in common cancers, like H3K27M in melanoma and AML.^196^

One of the most studied cancer-associated “oncohistones” carries the H3K27M substitution, whereby H3 lysine 27 is mutated to methionine, a missense mutation showing high genetic penetrance in pediatric glioblastomas^197,198^ (Fig. 5c). It is noteworthy that different H3 mutants are found in distinct locations. Indeed, *H3F3A* mutations such as H3.3K27M or H3.3G34R/V are found respectively in midline pediatric high-grade gliomas and cortex, whereas *HIST1H3B* mutations affecting the canonical H3.1 are restricted to the brainstem.^199^ *H3F3A* which encodes the histone variant H3.3 is found mutated in 60% of Diffuse Intrinsic Pontine Glioma (DIPG) cases^198^ and this mutation is suggested to be the first hit in DIPG tumorigenesis.^200^ This driver mutation is associated with obligate partner mutations throughout tumor progression,^200^ in particular in the cell cycle regulatory gene *TP53* or the chromatin remodeler *ATRX*. Interestingly, while H3.3K27M represents less than 10% of total H3, this level is sufficient to induce a significant decrease in the trimethylated state of H3K27, leading to a decrease in PcG-dependent transcriptional silencing.^201,202^

The epigenome is drastically altered in an H3K27M context. Indeed, while H3K27me3 is specifically restricted to unmethylated CGIs and H3K27me3/2 levels are significantly decreased, the monomethylation level of H3K27 remains unchanged.^203^ Intriguingly, H3K27me1 distribution is completely rewired in an H3K27M context.^203^ Moreover, just like in a wild-type H3 context, H3K36me2 restricts the spreading of H3K27me2/3.^203^ Furthermore, H3K27Ac levels are globally increased at the H3K27M location.^201,204^ This suggests that H3K27M has a dominant negative effect on the catalytic activity of the EZH2 methyltransferase.^201,202^

There is a strong interest in understanding the molecular mechanisms by which oncohistone mutations change the epigenome and impact gene expression. PRC2 was proposed to have a higher affinity for the mutated histone, which binds the EZH2 enzymatic domain, inhibiting its methyltransferase activity.^201,205,206^ However, the mechanism by which H3K27M oncohistones inhibits PRC2 activity is still under debate.^207^ The finding that PRC2 appears to be excluded from the H3K27M-K27Ac domains^208^ argues against the model of PRC2 sequestration by H3K27M. Moreover, while it is suggested that gliomagenesis is dependent on PRC2 inhibition,^201^ it has been demonstrated that loss of PRC2 disables growth and colony formation in H3K27M-positive DIPG cells, underlying the importance of PRC2 in tumor maintenance.^208^

Interestingly, CATACOMB/EZHIP, a PRC2 co-factor, either via its overexpression or a chromosomal translocation inducing its fusion with the NuA4 subunit gene *MBTD1*, described in low-grade endometrial stromal sarcoma,^209^ decreases PRC2-dependent methyltransferase activity.^9^ CATACOMB/EZHIP-dependent hypomethylation is due to a conserved methionine residue M406 which inhibits EZH2, mimicking the H3K27M oncohistone.^9^ Moreover, H3K27M and CATACOMB/EZHIP are mutually exclusive in gliomas, specifically in Posterior Fossa A (PFA) ependymomas.^210^ Both are suggested to decrease H3K27 trimethylation by blocking the spreading of the repressive mark from CGIs.^211^

In Giant Cell Tumor of the bone (GCT), the oncohistone H3.3G34W is encountered in 90% of cases.^212^ Interestingly, this residue is not post-translationally modified but its impact on the epigenome is undeniable. This mutation leads to loss of H3K36me3 which counteracts H3K27me3 deposition by PRC2.^212^ As a consequence, a redistribution of the H3K27me3 repressive mark occurs from intergenic to genic regions, resulting in perturbation in Polycomb-mediated silencing and in the maintenance of a progenitor state of the mutated cells.^212^

As previously mentioned, a crosstalk exists between H3K36me2/3 and H3K27me3 and this interplay remains in the presence of the H3K36M oncohistones, in which lysine 36 of the histone 3 is replaced by a methionine. This mutation is found in 95% of chondroblastomas and 92% of GCT, respectively in the *H3F3B* and *H3F3A* genes.^213^ Following the oncohistone paradigm, the H3K36me2/3 PTMs are reduced due to the inability of specific methyltransferases, namely SETD2, NSD1-NSD3, to deposit their marks.^214,215^ H3K36M reduces H3K36 methylation and increases nucleosome availability for PRC2 to deposit H3K27me3.^214^ The genome-wide increase in this repressive mark then induces a PRC1 redeployment which overall dilutes PRC1 at its canonical binding sites, leading to de-repression of self-renewal genes^214,216^ (Fig. 5c). Similarly, in human papillomavirus (HPV)-negative head and neck squamous cell carcinomas (HNSCCs),^217^ the H3K36 methylation state is involved in oncogenic promotion.^217^ NSD1 writer mutations, similarly to H3K36M, remodel the chromatin landscape by decreasing H3K36me2 levels. Considering the interplay between H3K36me2 and H3K27me3, Polycomb components would be expected to be involved in HNSCCs. However, the precise mechanism at play is yet to be characterized.

All these data show that the emerging oncohistone field is an important area of oncology, but much remains to be done and a current strong focus is on the investigation of how histone mutations contribute to epigenome reprogramming and whether these mutations are primarily drivers or contributors of tumorigenesis in a wide range of human cancers.^194^

### Non-genetic drug resistance in cancer

The ability of cancer cells to adapt to or resist anti-cancer therapies may be inherently of a genetic nature or may be acquired during treatment.^218^ Alongside an undergoing genetic evolution of cancer genomes, cancer cells can also be modified in their epigenetic landscapes and this non-genetic contribution can play a major role in cancer resistance. In fact, relapsing patients often do not present specific mutations that would explain a lower efficiency for the same therapy.^219,220^

Cancer cells are actually able to evolve and change completely their transcriptional landscape to adapt to treatment-induced stress. Polycomb implication in cancer drug-resistance depends on PRC2 and its catalytic activity as well as on other concomitant mechanisms that can induce transcriptional plasticity.^221–223^

In multiple myeloma (MM), cell adhesion-mediated drug resistance (CAM-DR) develops when malignant plasma cells interact with stromal cells in the bone marrow and become less sensitive to chemotherapy.^224^ In an in vitro system that recapitulates CAM-DR, anti-MM treatment results in an increase and redistribution of H3K27me3 in a dose-dependent manner in cultured MM cells only when they do not adhere to stromal cells.^224^ CAM-DR counteracts drug-induced H3K27 hypermethylation via phosphorylation of EZH2 at serine 21, leading to overexpression of anti-apoptotic genes which participate in survival and drug-resistance.^224^ In addition, *miR-15a* downregulation triggers PHF19 upregulation in relapsed MM patients.^225^ The involvement of PHF19 in drug-resistance might depend on its ability to stimulate proliferation by promoting EZH2 serine 21 phosphorylation, which inhibits the H3K27me3 deposition and leads to upregulation of genes linked to cell growth.^225^

In Testicular Germ Cell Tumors (TGCT), resistance to cisplatin is accompanied by a global decrease in H3K27me3 and H2AK119Ub levels, leading to upregulation of Polycomb target genes.^223^ Inhibition of the UTX and JMJD3 enzymes, responsible for H3K27 demethylation, is sufficient to increase H3K27me3 and make TGCT cells more sensitive to the initial chemotherapy.^223^

In AML, therapeutic resistance can arise in the apparent absence of new genetic mutations and is antagonized by inhibiting Lsd1, a demethylase chromatin modulator involved in the regulation of enhancer activity.^219^ Inhibition of Lsd1 creates enhancer switching, generating new binding sites for pioneer factors that ultimately activate the enhancers of key drug resistance genes. Inhibition of a key chromatin modulator in AML then makes it possible to resensitize cells to the primary treatment.^219^

Unlike mutations, failed or disrupted epigenetic mechanisms can be quite easily reverted using epidrugs to overcome cancer progression by rewiring malignant epigenomes, either to resensitize tumor cells resistant to conventional therapy or to sensitize them to new therapies. Given the importance of PcG proteins in transcriptional regulation, it will therefore be of great interest to further characterize the mechanisms by which PcG proteins contribute to drug-resistance. In particular, it will be important to expand research aimed at understanding Polycomb functions at enhancers,^139,226^ since they might be involved in various cancer types and stages.

## Conclusive remarks: Polycomb epigenetics in cancer

Although it is commonly assumed that cancer arises from a set of multiple mutations, a pan-cancer analysis established that about 5% of cancer cases did not have driver mutations that could explain tumorigenesis, pointing out that genetics might not be the only player in cancer.^227^ Non-genetic alterations appear to represent an alternative path toward the development, progression and drug-resistance of cancer cells. In pancreatic ductal adenocarcinoma, metastases do not show driver gene mutations but rather follow drastic epigenomic reprogramming,^181,228^ suggesting that epigenetic modifiers are mainly involved. Additionally, ependymomas — a childhood brain tumor — are characterized by a very low mutation rate,^229^ suggesting that cancer is not only a consequence of DNA mutations, but rather emerges and evolves from a crosstalk between genetic and non-genetic processes. In an extreme view, cancer has been defined as an “epigenetic disease”.^230^ It would therefore not be surprising to find misregulated Polycomb proteins as epi-drivers in tumorigenesis.

PcG proteins have imposed themselves in a wide range of biological processes. Clearly, they are landmark components in the field of cancer research and we have only started to understand the extent of their oncogenic functions. As most research focuses on the EZH2 catalytic subunit of PRC2, it will be interesting to better characterize the involvement of the different PRC2 subunits as well as on the many flavors of PRC1 complexes. Nonetheless, a fascinating part of the oncogenic function of PcG components relies on the fact that some of them can act both in a manner dependent or independent on Polycomb complexes.^231^ It will be interesting to better characterize these PcG functions at the molecular level in order to have a complete picture of their mode of action. While it is clear that the overexpression or downregulation of PcG proteins is involved in cancer, it will be important to characterize how modifying protein stability by either PTMs and/or interaction with yet unidentified partners might be implicated in tumorigenesis. Finally, context is of paramount importance: misregulation of Polycomb proteins results in different, sometimes even opposing results in different cancer types. Identifying molecular pathways leading to these context-dependent effects will be crucial in order to improve cancer diagnosis, prognosis and therapy.
